# Quantification of myocardial strain in patients with isolated left ventricular non-compaction and healthy subjects using deformable registration algorithm: comparison with feature tracking

**DOI:** 10.1186/s12872-020-01668-x

**Published:** 2020-09-03

**Authors:** Jia Liu, Yumin Li, Yue Cui, Yukun Cao, Sheng Yao, Xiaoyue Zhou, Jens Wetzl, Wenjuan Zeng, Heshui Shi

**Affiliations:** 1 Department of Radiology, Union Hospital, Tongji Medical College, Huazhong University of Science and Technology, Wuhan, 430022 China; 2 Hubei Province Key Laboratory of Molecular Imaging, Wuhan, 430022 China; 3 Department of Orthopedics, Union Hospital, Tongji Medical College, Huazhong University of Science and Technology, Wuhan, 430022 China; 4 MR Collaboration, Siemens Healthineers Ltd., Shanghai, China; 5 Siemens Healthcare, Erlangen, Germany; 6 Clinical Laboratory, Union Hospital, Tongji Medical College, Huazhong University of Science and Technology, Wuhan, 430022 China

**Keywords:** Isolated left ventricular non-compaction, Cardiac magnetic resonance, Myocardial strain, Deformable registration algorithm, Feature tracking, Ejection fraction, Reproducibility

## Abstract

**Background:**

Systolic dysfunction of the left ventricle is frequently associated with isolated left ventricular non-compaction (iLVNC). Clinically, the ejection fraction (EF) is the primary index of cardiac function. However, changes of EF usually occur later in the disease course. Feature tracking (FT) and deformable registration algorithm (DRA) have become appealing techniques for myocardial strain assessment.

**Methods:**

Thirty patients with iLVNC (36.7 ± 13.3 years old) and fifty healthy volunteers (42.3 ± 13.6 years old) underwent cardiovascular magnetic resonance (CMR) examination on a 1.5 T MR scanner. Strain values in the radial, circumferential, longitudinal directions were analyzed based on the short-axis and long-axis cine images using FT and DRA methods. The iLVNC patients were further divided based on the ejection fraction, into EF ≥ 50% group (*n* = 11) and EF < 50% group (*n* = 19). Receiver-operating-characteristic (ROC) analysis was performed to assess the diagnostic performance of the global strain values. Intraclass correlation coefficient (ICC) analysis was used to evaluate the intra- and inter-observer agreement.

**Results:**

Global radial strain (GRS) was statistically lower in EF ≥ 50% group compared with control group [GRS (DRA)/% vs. controls: 34.6 ± 7.0 vs. 37.6 ± 7.2, *P* < 0.001; GRS (FT)/% vs. controls: 37.4 ± 13.2 vs. 56.9 ± 16.4, *P* < 0.01]. ROC analysis of global strain values derived from DRA and FT demonstrated high area under curve (range, 0.743–0.854). DRA showed excellent intra- and inter-observer agreement of global strain in both iLVNC patients (ICC: 0.995–0.999) and normal controls (ICC: 0.934–0.996). While for FT analysis, global radial strain of normal controls showed moderate intra-observer (ICC: 0.509) and poor inter-observer agreement (ICC: 0.394).

**Conclusions:**

In patients with iLVNC, DRA can be used to quantitatively analyze the strain of left ventricle, with global radial strain being an earlier marker of LV systolic dysfunction. DRA has better reproducibility in evaluating both the global and segmental strain.

## Background

Left ventricular non-compaction (LVNC) is an uncommon cardiomyopathy characterized by excessively prominent trabeculations and deep inter-trabecular recesses. The thickened wall of left ventricle (LV) consists of a thin compacted epicardial layer and a thick non-compacted endocardial layer. This disease is likely to result from an arrest of normal embryogenesis, mainly involving the endocardium and myocardium [1]. It may develop in isolation (isolated left ventricular non-compaction, iLVNC) or accompanied by congenital heart disease [2].

Cardiac magnetic resonance (CMR) imaging is considered as the standard of reference to clinically assess volumes and global systolic function of left ventricle [2]. LV systolic dysfunction is frequently associated with iLVNC [3]. Clinically, left ventricular ejection fraction (LVEF) is the most commonly used indicator for evaluating cardiac function. However, changes in the LVEF usually occur late in the disease course [4]. Besides, the wall motion is usually evaluated on a subjective basis and the accuracy may depend on the experience of the rater. Previous studies have shown that myocardial strain may facilitate early detection and outcome assessment of some cardiac diseases [5, 6]. The term “strain” is defined as the change in fiber length divided by the original length, and can be quantified in three directions of myocardial fiber (radial, circumferential, and longitudinal) [7]. Several post-processing methods have been applied using standard steady state free precession (SSFP) cine images, such as deformable registration algorithm (DRA) and feature tracking (FT). DRA and FT can evaluate both global and regional myocardial function in volunteers and patients [8, 9]. Recent FT studies indicated that global strain values based on CMR were impaired in LVNC patients [10, 11]. Up to date, DRA technique has not been applied to quantify myocardial strain in patients with iLVNC.

In the present study, we aimed to 1) investigate the role of strain assessment in detection of early dysfunction of left ventricle in patients with iLVNC, 2) assess the accuracy of global strain to distinguish iLVNC from healthy people, and 3) compare the reproducibility of global and segmental strains derived from DRA and FT in healthy volunteers and iLVNC patients.

## Methods

### Study population

The diagnosis of LVNC was based on the presence of established CMR and clinical criteria: 1) a thin, compacted (C) epicardial layer and a thick, non-compacted (NC) endocardial layer with prominent trabeculations and deep intertrabecular recesses; 2) NC/C ratio measured at end-diastole > 2.3 in at least one LV segment [12]. Exclusion criteria included: age < 18 years, significant valvular heart disease, clinical or CMR evidence of ischemic heart disease and evidence of concomitant congenital heart disease. Fifty age- and sex-matched healthy volunteers who responded to advertisements were recruited to participate in this study. Eventually, thirty patients with iLVNC (63.3% males, 36.7 ± 13.3 years old) and fifty healthy volunteers (58% males, 42.3 ± 13.6 years old) were included.

### CMR imaging acquisition

All subjects underwent a standard CMR examination with a 1.5 T scanner (MAGNETOM Aera, Siemens Healthcare, Erlangen, Germany). A stack of short-axis views and three long-axis (2-, 3-, and 4-chamber) views were acquired. The whole LV was imaged from the base to the apex with the following parameters: repetition time (TR)/echo time (TE) of 2.9/1.2 ms, slice thickness of 6 mm, matrix of 186 × 256 pixels, and flip angle of 80°.

### CMR image analysis

#### Cardiac function

A dedicated workstation employing commercial software (Argus, Siemens Healthcare, Erlangen, Germany) was used to analyze LV volume and function. Cardiac volumetric and functional parameters were quantified based on manual delineation of the endocardial and epicardial borders using a stack of continuous short-axis cine images. The trabeculae and papillary muscles were included in the left ventricular cavity. The end-diastolic volume (EDV), end-systolic volume (ESV), stroke volume (SV), ejection fraction (EF) were measured and indexed to the patient’s body surface area (BSA).

#### Strain

**Fig. 1:**
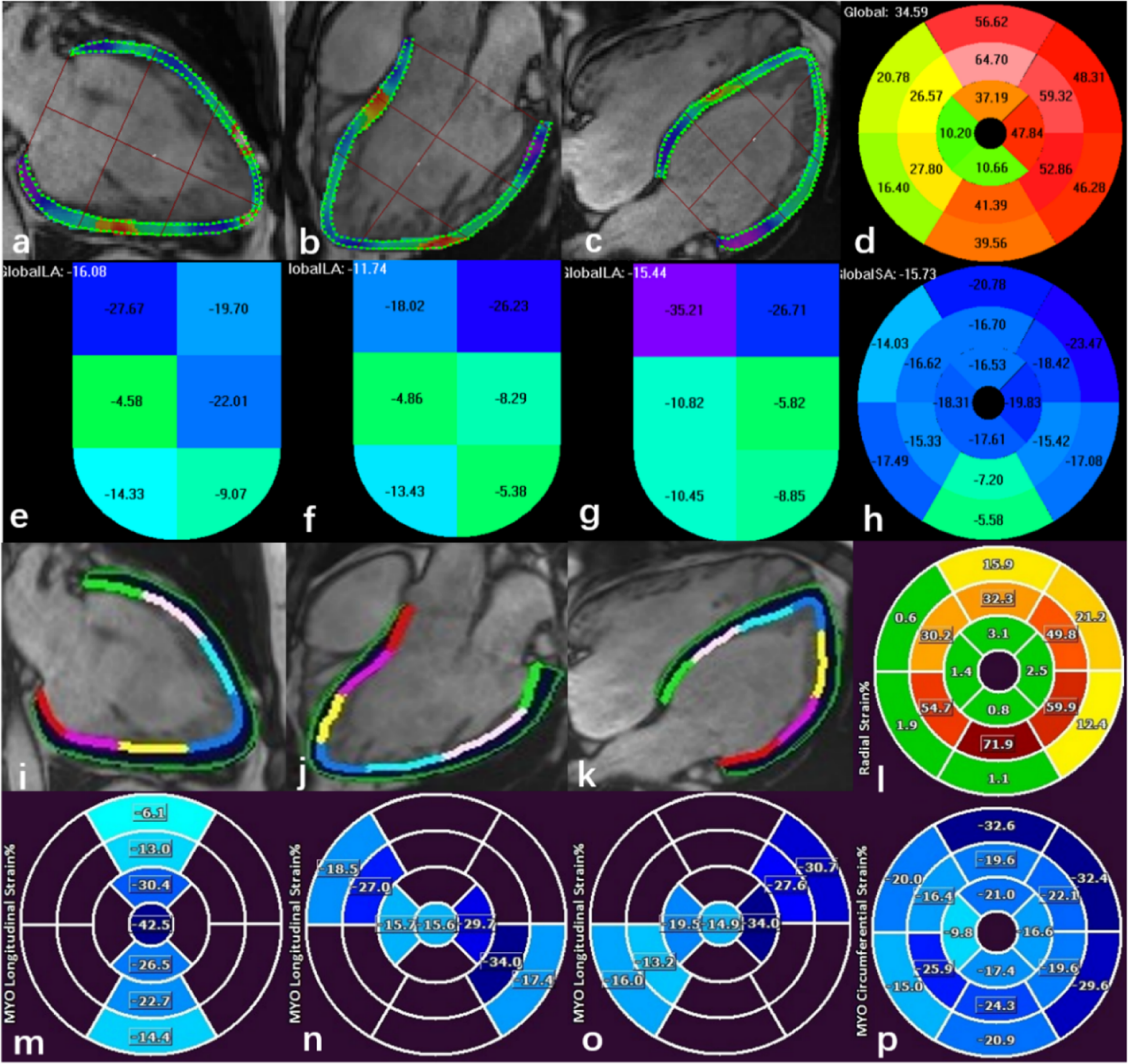
Representative example of a patient with iLVNC. Measurement diagram of the peak systolic strain value of the left ventricular myocardium using DRA (**a**-**h**) and FT (**i**-**p**) in the same patient. Images **a**-**c**, **i**-**k** show the tracking contours of longitudinal strain based on the long axis in the 2-, 3- and 4-chamber view using DRA and FT, respectively. Images **e**-**g**, **m**-**o** show the segmental longitudinal strain values of the previous three images, respectively. Images **d** and **l** show the strain values of 16 segments in the radial direction using DRA and FT, respectively, which are derived from the short axis view. Images **h** and **p** show the strain values in the circumferential direction using DRA and FT, respectively, which are also derived from the short axis view

Cine bSSFP-based peak systolic strains were analyzed using a prototype DRA software (TrufiStrain, version 2.0, Siemens Healthcare, Erlangen, Germany) and FT software (Medis Suite, version 3.0, Medis Medical Imaging Systems, Leiden, the Netherlands). For FT analysis, we delineated the endocardial and epicardial contours manually at end-systole and adjusted them at end-diastole. For DRA analysis, the contours were drawn manually at end-diastole; then the consecutive contours on the other phases were tracked automatically through the cardiac cycle (Fig. 1). Detailed analysis with DRA has been described previously [13]. The global longitudinal strain (GLS) was calculated based on the average of the peak systolic strain values measured by the three long axis orientations. Short-axis views of the basal, mid-ventricular, and apical levels were used to analyze radial and circumferential strain. After a reference point was set up at the anterior insertion site, segmental circumferential strain and radial strain were calculated based on the 16-segment model. Finally, the peak systolic global and segmental myocardial strains were obtained for analysis.

### Statistical analysis

SPSS (version 22.0; Chicago, IL, USA) and MedCalc (version 19.0.7; Ostend, Belgium) software was used to perform the statistical analyses. The Anderson–Darling test was used to check the normality. Continuous data were expressed as the mean ± standard deviation. Means of two continuous normally distributed variables were compared by independent samples Student’s t-test. Other comparisons were performed using the Mann–Whitney U test. Pairwise comparisons between groups were performed using one-way ANOVA (normal distribution) or Kruskal–Wallis test with Bonferroni correction (non-normal distribution).

Receiver-operator-characteristic (ROC) analyses of global strains determined by DRA and FT were performed to assess the diagnostic capability of strain parameters in distinguishing iLVNC from healthy volunteers. The comparisons of areas under the curve (AUC) were performed using Delong test [14]. Correlation analysis between two parameters used the Spearman rank correlation test or Pearson correlation analysis.

For strain analysis, reproducibility was tested by using the intraclass correlation coefficient (ICC) on 15 healthy volunteers and 15 iLVNC patients. Inter-observer variability was analyzed on the same image set by two independent investigators (J.L., YM.L.). Intra-observer variability was analyzed on the same image set by one investigator (J.L.) with two readings 2 weeks apart. Based on the 95% confidence interval of the ICC estimate, values less than 0.50, between 0.50 and 0.75, between 0.75 and 0.90, and greater than 0.90 were indicative of poor, moderate, good, and excellent reliability, respectively [15].

## Results

### Demographic and clinical characteristics

All demographic and cardiac MRI characteristics are shown in Table 1.

**Table 1:** Clinical characteristics and parameters of cardiac function and myocardial deformation

Parameters	iLVNC (*n* = 30)	Controls (*n* = 50)	*P* value
Age (years)	36.7 ± 13.3	42.3 ± 13.6	0.101
Male (%)	19 (63.3%)	29 (58%)	0.637
BMI (kg/m^2^)	23.2 ± 3.7	22.8 ± 2.9	0.588
HR (bpm)	66.7 ± 9.0	65.3 ± 9.2	0.249
LVEF (%)	42.5 ± 14.3	57.7 ± 4.7	**< 0.001**
EDVI (ml·m^− 2^)	79.5 ± 44.8	69.8 ± 13.3	0.827
ESVI (ml·m^− 2^)	49.1 ± 40.0	29.9 ± 7.4	**0.016**
SVI (ml·m^− 2^)	30.4 ± 12.4	39.9 ± 7.4	**< 0.001**
CI (L·min^− 1^·m^− 2^)	2.0 ± 0.8	2.6 ± 0.5	**< 0.001**
NoNC	4.0 ± 2.2	–	–
GLS (DRA)/%	−11.9 ± 3.9	− 14.5 ± 1.4	**0.001**
GRS (DRA)/%	26.3 ± 11.0	37.6 ± 7.2	**< 0.001**
GCS (DRA) /%	−12.1 ± 4.2	−16.4 ± 1.6	**< 0.001**
GLS (FT)/%	−17.8 ± 6.4	− 23.3 ± 2.8	**< 0.001**
GRS (FT)/%	33.2 ± 23.5	56.9 ± 16.4	**< 0.001**
GCS (FT) /%	−15.5 ± 6.6	−22.5 ± 2.6	**< 0.001**

*BMI* Body mass index, *HR* Heart rate, *LVEF* Left ventricular ejection fraction, *EDVI* End-diastolic volume index, *ESVI* End-systolic volume index, *SVI* Stroke volume index, *CI* Cardiac index, *NoNC* Number of non-compacted segment, *DRA* Deformable registered algorithm, *FT* Feature tracking, *GRS* Global radial strain, *GCS* Global circumferential strain, *GLS* Global longitudinal strain

### Correlations between LVEF and global strain of iLVNC patients

**Fig. 2:**
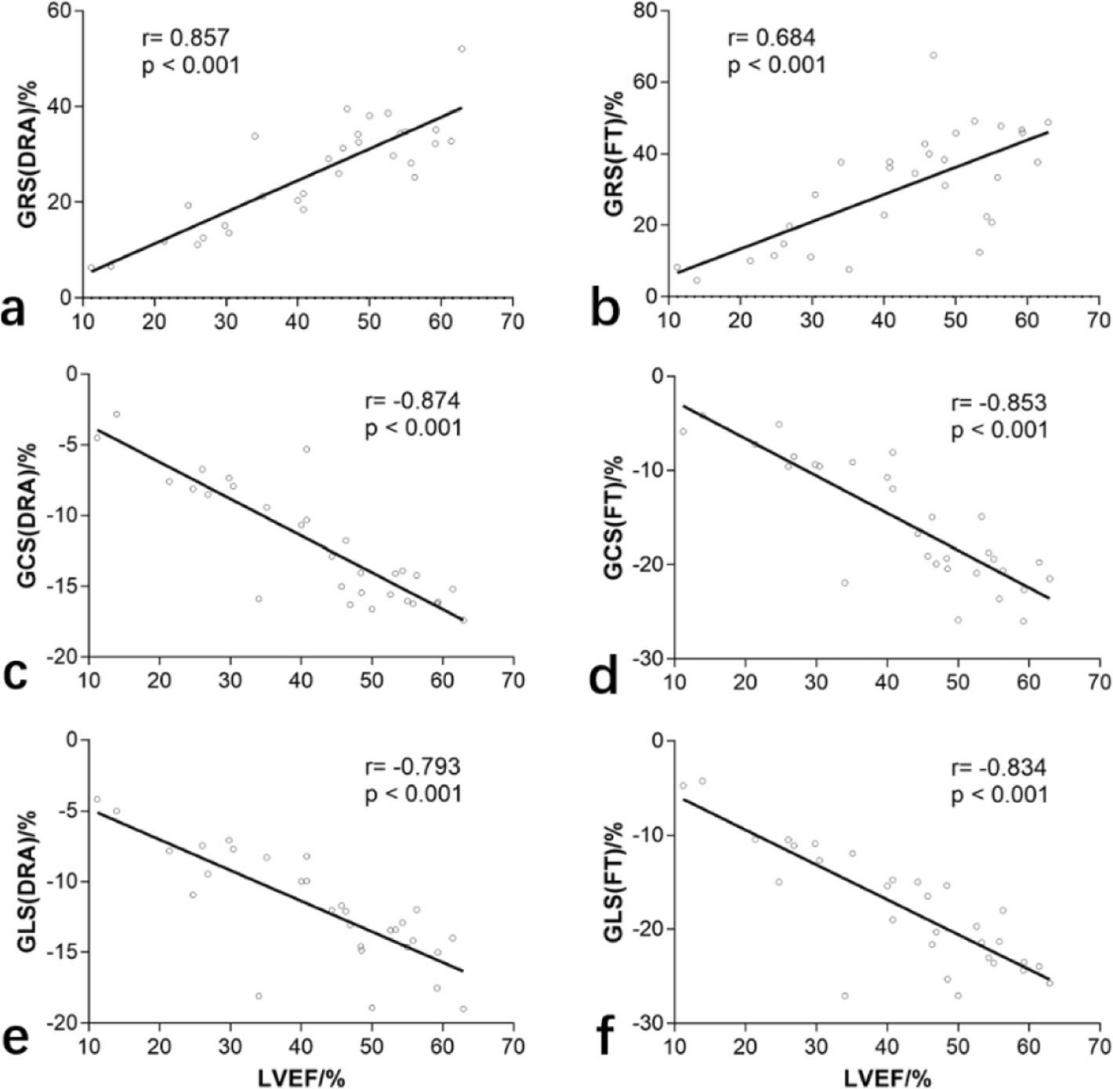
Correlation between global strain values (derived from DRA and FT) and LVEF. Significant positive correlations between GRS and LVEF (image **a** and **b**), and significant negative correlations between GCS and LVEF (image **c** and **d**), and GLS and LVEF (image **e** and **f**) are evident

Correlations between left ventricular ejection fraction (LVEF) and global strain values derived from DRA and FT in iLVNC patients are shown in Fig. 2. All global strain values showed significant correlations with LVEF (*P* < 0.001 for all).

### Comparison of strain values derived from DRA and FT among iLVNC patients with EF ≥ 50% group, EF < 50% group, and control group

As shown in Table 2, global radial strain (GRS) values derived from DRA and FT were significantly lower in iLVNC patients with EF ≥ 50% than those in control group, whereas global circumferential strain (GCS) and global longitudinal strain (GLS) values didn’t show significant difference between iLVNC patients with EF ≥ 50% and normal subjects.

**Table 2:** Comparison between iLVNC patients with EF ≥ 50%, EF < 50% and normal controls

Parameters	iLVNC (*n* = 30)		Controls (*n* = 50)
	EF ≥ 50% (*n* = 11)	EF < 50% (*n* = 19)	
GRS (DRA)/%	34.6 ± 7.0*	21.4 ± 10.0*	37.6 ± 7.2
GCS (DRA)/%	−15.6 ± 1.1	−10.0 ± 4.0*	− 16.4 ± 1.6
GLS (DRA)/%	−14.0 ± 2.4	− 10.1 ± 3.5*	−14.5 ± 1.4
GRS (FT)/%	37.4 ± 13.2#	26.6 ± 16.3*	56.9 ± 16.4
GCS (FT)/%	−21.3 ± 3.2	−12.2 ± 5.7*	−22.5 ± 2.6
GLS (FT)/%	− 22.9 ± 2.6	−14.8 ± 6.0*	−23.3 ± 2.8
EF	56.4 ± 3.9	34.5 ± 11.6*	57.7 ± 4.7

#*P* < 0.01 vs. controls, *P < 0.001 vs. controls

### Distinction of iLVNC patients and normal subjects

The results of ROC analyses using global strains to distinguish iLVNC patients from normal controls are shown in Table 3. ROC analysis using GLS, GRS, and GCS derived from DRA and FT all demonstrated high AUC values. However, on pairwise comparison of ROC curves, there were no statistically significant differences (*P* > 0.05).

**Table 3:** Accuracy of the global strains (GLS, GRS and GCS) derived from two methods (DRA and FT) for the distinction of iLVNC

	Sensitivity (%)	Specificity (%)	AUC	Cutoff value	95% CI
GLS					
DRA	76.7	68.0	0.743	−13.7	0.616–0.871
FT	60.0	100.0	0.794	−18.9	0.680–0.908
GRS					
DRA	68.0	90.0	0.850	35.0	0.765–0.935
FT	88.0	73.3	0.854	40.1	0.761–0.947
GCS					
DRA	60.0	86.0	0.749	−14.8	0.631–0.868
FT	70.0	80.0	0.818	−20.0	0.715–0.921

*AUC* Area under the curve, *CI* Confidence interval

### Intra-observer and inter-observer reproducibility of global strains

Intra- and inter-observer agreements of global myocardial strain for DRA and FT are shown in Table 4. DRA showed excellent intra- and inter-observer agreement in both iLVNC patients and normal controls. While for FT analysis, GRS of normal controls showed moderate intra-observer and poor inter-observer agreement. The intra- and inter-observer agreements of global strain derived from FT performed in iLVNC patients were higher than those in normal controls, especially the GRS.

**Table 4:** Reproducibility of the global strains (GLS, GRS and GCS) derived from two methods (DRA and FT) using intraclass correlation coefficient analysis

	Controls		iLVNC	
	DRA	FT	DRA	FT
GRS				
inter	0.996^a^	0.394	0.999^a^	0.716
intra	0.996^a^	0.509	0.996^a^	0.785^a^
GCS				
inter	0.934^a^	0.891^a^	0.996^a^	0.986^a^
intra	0.958^a^	0.843^a^	0.995^a^	0.982^a^
GLS				
inter	0.982^a^	0.891^a^	0.995^a^	0.976^a^
intra	0.977^a^	0.879^a^	0.997^a^	0.980^a^

*intra* Intra-observer agreement, *inter* Inter-observer agreement

^a^ Intraclass correlation coefficient>75%

### Intra-observer and inter-observer reproducibility of segmental strains

As shown in Table 5, DRA showed better observer agreement for segmental strains compared with FT in both healthy volunteers and patients.

**Table 5:** Reproducibility of segmental strains (RS, CS and LS) using two methods (DRA and FT) in controls and iLVNC patients

Segment		Controls						iLVNC					
		DRA			FT			DRA			FT		
		RS	CS	LS	RS	CS	LS	RS	CS	LS	RS	CS	LS
1	inter	0.984^a^	0.866^a^	0.714	0.266	0.747	0.702	0.720	0.704	0.951^a^	0.312	0.704	0.886^a^
	intra	0.984^a^	0.919^a^	0.991^a^	0.629	0.782^a^	0.744	0.978^a^	0.959^a^	0.939^a^	0.265	0.578	0.919^a^
2	inter	0.713	0.870^a^	0.989^a^	0.448	0.600	0.804^a^	0.979^a^	0.746	0.993^a^	0.561	0.746	0.532
	intra	0.804^a^	0.902^a^	0.996^a^	0.752^a^	0.661	0.820^a^	0.936^a^	0.794^a^	0.996^a^	0.233	0.649	0.891^a^
3	inter	0.984^a^	0.933^a^	0.922^a^	0.709	0.612	0.771^a^	0.730	0.823^a^	0.969^a^	0.619	0.823^a^	0.853^a^
	intra	0.989^a^	0.989^a^	0.936^a^	0.802^a^	0.766^a^	0.806^a^	0.932^a^	0.962^a^	0.984^a^	0.618	0.821^a^	0.745
4	inter	0.706	0.909^a^	0.940^a^	0.369	0.786^a^	0.578	0.912^a^	0.860^a^	0.979^a^	0.718	0.860^a^	0.946^a^
	intra	0.740	0.951^a^	0.971^a^	0.683	0.904^a^	0.694	0.996^a^	0.989^a^	0.978^a^	0.876^a^	0.805^a^	0.916^a^
5	inter	0.686	0.910^a^	0.924^a^	0.174	0.771^a^	0.809^a^	0.913^a^	0.427	0.970^a^	0.775^a^	0.427	0.923^a^
	intra	0.739	0.920^a^	0.958^a^	0.435	0.800^a^	0.841^a^	0.998^a^	0.993^a^	0.965^a^	0.503	0.389	0.560
6	inter	0.880^a^	0.915^a^	0.962^a^	0.164	0.861^a^	0.935^a^	0.883^a^	0.274	0.914^a^	0.423	0.274	0.854^a^
	intra	0.892^a^	0.922^a^	0.989^a^	0.337	0.893^a^	0.956^a^	0.996^a^	0.858^a^	0.982^a^	0.173	0.338	0.688
7	inter	0.967^a^	0.956^a^	0.976^a^	0.337	0.809^a^	0.761^a^	0.951^a^	0.914^a^	0.955^a^	0.576	0.914^a^	0.934^a^
	intra	0.980^a^	0.979^a^	0.981^a^	0.403	0.881^a^	0.798^a^	0.998^a^	0.982^a^	0.978^a^	0.491	0.894^a^	0.868^a^
8	inter	0.915^a^	0.827^a^	0.784^a^	0.133	0.554	0.681	0.929^a^	0.883^a^	0.964^a^	0.074	0.883^a^	0.543
	intra	0.936^a^	0.868^a^	0.680	0.303	0.656	0.752^a^	0.994^a^	0.528	0.956^a^	0.428	0.627	0.854^a^
9	inter	0.740	0.978^a^	0.980^a^	0.049	0.850^a^	0.558	0.695	0.979^a^	0.988^a^	0.742	0.979^a^	0.901^a^
	intra	0.735	0.975^a^	0.976^a^	0.509	0.875^a^	0.776^a^	0.823^a^	0.982^a^	0.991^a^	0.756^a^	0.943^a^	0.898^a^
10	inter	0.939^a^	0.774^a^	0.955^a^	0.100	0.643	0.827^a^	0.948^a^	0.923^a^	0.981^a^	0.328	0.923^a^	0.949^a^
	intra	0.951^a^	0.843^a^	0.945^a^	0.098	0.678	0.850^a^	0.964^a^	0.977^a^	0.921^a^	0.475	0.823^a^	0.920^a^
11	inter	0.839^a^	0.910^a^	0.982^a^	0.292	0.716	0.856^a^	0.980^a^	0.916^a^	0.978^a^	0.733	0.916^a^	0.956^a^
	intra	0.842^a^	0.945^a^	0.980^a^	0.294	0.718	0.896^a^	0.992^a^	0.985^a^	0.989^a^	0.689	0.711	0.759^a^
12	inter	0.976^a^	0.794^a^	0.985^a^	0.144	0.580	0.691	0.934^a^	0.944^a^	0.976^a^	0.658	0.944^a^	0.867^a^
	intra	0.981^a^	0.867^a^	0.989^a^	0.310	0.586	0.710	0.994^a^	0.963^a^	0.994^a^	0.708	0.892^a^	0.723
13	inter	0.980^a^	0.830^a^	0.991^a^	0.334	0.890^a^	0.893^a^	0.579	0.784^a^	0.994^a^	0.426	0.784^a^	0.908^a^
	intra	0.981^a^	0.861^a^	0.991^a^	0.367	0.912^a^	0.898^a^	0.988^a^	0.975^a^	0.995^a^	0.541	0.819^a^	0.959^a^
14	inter	0.977^a^	0.957^a^	0.975^a^	0.189	0.909^a^	0.649	0.848^a^	0.660	0.986^a^	0.418	0.660	0.953^a^
	intra	0.989^a^	0.958^a^	0.984^a^	0.335	0.928^a^	0.685	0.995^a^	0.447	0.961^a^	0.563	0.887^a^	0.834^a^
15	inter	0.739	0.942^a^	0.975^a^	0.051	0.838^a^	0.816^a^	0.804^a^	0.931^a^	0.964^a^	0.352	0.931^a^	0.840^a^
	intra	0.791^a^	0.951^a^	0.992^a^	0.095	0.868^a^	0.906^a^	0.984^a^	0.960^a^	0.993^a^	0.638	0.936^a^	0.682
16	inter	0.786^a^	0.796^a^	0.951^a^	0.008	0.812^a^	0.885^a^	0.951^a^	0.938^a^	0.997^a^	0.426	0.938^a^	0.954^a^
	intra	0.808^a^	0.846^a^	0.965^a^	0.024	0.908^a^	0.886^a^	0.998^a^	0.325	0.989^a^	0.552	0.881^a^	0.935^a^

*intra* Intra-observer agreement, *inter* Inter-observer agreement

^a^ Intraclass correlation coefficient>75%

## Discussion

Our study found that global radial strain derived from DRA and FT was reduced in iLVNC patients with normal EF. Global strain values had high diagnostic capability to distinguish iLVNC from healthy volunteers. Compared with FT, DRA showed better intra- and inter-observer agreement for global and segmental strains in both normal controls and iLVNC patients.

In our study, global strains derived from both DRA and FT had good correlation with the LVEF. Compared to EF which acts as a global functional parameter, strain reveals the microscopic view of myocardial function. Murphy and colleagues have stated that there is a long preclinical phase of disease during which patients have no symptoms or are pauci-symptomatic [16]. A previous study pointed out that patients with EF < 50% were eligible for drug treatment [17]. In our study, patients with iLVNC were further divided into two groups based on the ejection fraction (EF): EF ≥ 50% and EF < 50%. We found that GRS values derived from DRA and FT were significantly reduced in iLVNC patients with EF ≥ 50%. This indicates that GRS may detect early left ventricular dysfunction in iLVNC patients with normal EF. As previous studies have proposed, the compaction progress of the myocardium develops from the epicardium to the endocardium and from the ventricular base to the apex; therefore, arrest of the compaction process is prone to occur in the apical endocardium of the ventricle [18, 19]. The left ventricular apex has a great component of radially and circumferentially oriented myocardial fibers, rather than longitudinally oriented fibers, while circumferential myofibers typically distribute in the epicardial region of LV wall [20]. The differences of anatomy may help understand the potentially superior clinical significance of radial strain in iLVNC patients.

In our study, all three global strains by the DRA and FT had high AUC values (0.743 and higher) in distinguishing iLVNC from healthy volunteers. However, there was no statistically significance difference of the AUC values between the two methods in any orientation. As mentioned, arrest of the compaction process is prone to occur in the apical endocardium of the ventricle [18]. However, in real situation, the distribution of the trabeculae may be random and complex. In addition, the strain reduction may further aggravate in the range and extent as the disease progresses, hence the global strains may be affected in all directions with no significant differences.

When evaluating the robustness of a strain-analysis method, reproducibility is an important factor. In our study, the reproducibility analysis was performed in both iLVNC patients and volunteers. We found that the intra- and inter-observer agreements of global and segmental strains were better for DRA than FT in both normal controls and iLVNC patients, especially for radial strain analysis. This is probably because that DRA is a novel method to analyze cardiac strain on a pixel-by-pixel basis. Furthermore, DRA can automatically segment the left ventricle on CMR cine images. It helps obtain the deformation from any frame to any other frame in the cardiac cycle by recovering a set of both forward and backward deformation fields. Different segmentation contours can be formed on each phase in the cardiac cycle, and then propagated to other phases [21, 22]. Thereafter, the best combination of contour series is retained which makes the algorithm more efficient and time-saving. Although the FT algorithm has been developed for analyzing myocardial motion for a long time, applying it to clinical image data is challenging. After delineating the contours on one reliable image, the traced contours are propagated in time by identifying the same features in the following frames (optical flow technique). But some features may fade from one frame to another during tracking, and blood motion can interfere with the deformation computation [8]. It is very likely that small differences in manual contouring of endo- and epicardial borders between observers may have an important impact on radial strain measurements, while the impact on the reproducibility of longitudinal or circumferential strain is much smaller. A previous study also reported that reproducibility in DRA analysis was better than FT [23]. As mentioned above, the tracking signal sources are different between the two techniques, and readers need to adjust the contours when performing FT analysis, which is not needed when using DRA. The better reproducibility of DRA in both global and segmental view might be, more likely, related to the fully automated segmentation approach, as compared with FT [21]. A recent study comparing DRA and tagged CMR showed that the DRA demonstrated a strong relationship with the harmonic phase (HARP) for myocardial GLS (*R*^2^ = 0.75; *P* < 0.0001) and GCS (*R*^2^ = 0.61; *P* < 0.0001), and DRA demonstrated consistently lower coefficient of variation (CV) than tagged CMR [24].

A study using speckle tracking echocardiography indicated that apical circumferential strain may be a potential diagnostic tool for children with noncompaction cardiomyopathy (sensitivity: 87%, specificity: 79%, AUC: 0.88, *P* < 0.001) [25]. Since DRA showed better reproducibility in segmental strain in our study, we believe that it may be a good tool for evaluating the value of CMR-based segmental strain in diagnosing iLVNC patients in the future.

Meanwhile, we found that reproducibility of FT-based GRS performed in iLVNC patients was better than that in normal controls, while FT-based GCS and GLS both showed a good reproducibility in controls and iLVNC patients. The radial strain estimate heavily depends on how well the myocardial border is detected, and large variations were observed in radial strain [26]. As for the better reproducibility in iLVNC patients in our study, we assume that patients with iLVNC have thinner myocardium and weaker myocardial motion than normal people. Therefore, to some extent, the contour can be better and more accurately tracked by readers. In terms of absolute values, DRA-derived strain values were consistently lower (more negative) compared to FT, especially for radial strain values. Previous studies have demonstrated higher absolute values by FT compared to CMR tagging [27, 28]. Results of the DRA analysis in our study are within normal ranges of reported MR tagging data. Strain quantification methods differ considerably in their registration of myocardial motion, so it is not surprising that some techniques generate similar values [29, 30] while others do not [31, 32].

## Limitations

This study has some limitations that should be acknowledged. Patients with iLVNC were retrospectively enrolled, the limitations of which are well known. Additionally, our study was a single-center study and the sample size was relatively small. We also didn’t consider the possible combination of iLVNC with myocardial fibrosis.

## Conclusions

In conclusion, DRA can be used to quantitatively analyze the strain of left ventricle in patients with iLVNC, and GRS is an earlier marker than LVEF of LV systolic dysfunction. Global strains can distinguish patients with iLVNC from healthy subjects with high diagnostic accuracy. Compared with FT, DRA has an excellent reproducibility in evaluating both global and segmental strain values in iLVNC patients and healthy population. Therefore, DRA can be a reliable tool to clinically assess global and segmental myocardial function of iLVNC patients.

## Data Availability

The datasets used or analyzed during the current study are available from the corresponding author on reasonable request.

## Abbreviations

iLVNC: Isolated left ventricular non-compaction
DRA: Deformable registration algorithm
FT: Feature tracking
LVEF: Left ventricular ejection fraction
CMR: Cardiovascular magnetic resonance
SSFP: Standard steady state free precession
BSA: Body surface area
EDVI: Left ventricular end-diastolic volume index
ESVI: End-systolic volume index
SVI: Stroke volume index
CI: Cardiac index
GRS: Global radial strain
GCS: Global circumferential strain
GLS: Global longitudinal strain
ROC: Receiver-operator-characteristic
ICC: Intraclass correlation coefficient
AUC: Area under curve
